# Supplementary material for: Myogenetic Oligodeoxynucleotide Induces Myocardial Differentiation of Murine Pluripotent Stem Cells
Source: Int J Mol Sci. 2023 Sep 21;24(18):14380. doi: 10.3390/ijms241814380 (PMC10532123; doi:10.3390/ijms241814380)
Supplement: Supplementary file 1 [file ijms-24-14380-s001.zip › ijms-2570402-supplementary.pdf]

International Journal of
Molecular Sciences

MDPI

Supplementary Materials

Myogenetic Oligodeoxynucleotide Induces Myocardial Differentiation of Murine Pluripotent Stem Cells

Mina Ishioka [1], Yuma Nihashi [2], Yoichi Sunagawa [3], Koji Umezawa [4,5], Takeshi Shimosato [1,4,5], Hiroshi Kagami [4], Tatsuya Morimoto [3], and Tomohide Takaya [1,4,5,*]

[11] Department of Agriculture, Graduate School of Science and Technology, Shinshu University, 8304 Minami-minowa, Kami-ina, Nagano 399-4598, Japan
[2] Cellular and Molecular Biotechnology Research Institute, National Institute of Advanced Industrial Science and Technology, Central 5-41, 1-1-1 Higashi, Tsukuba 305-8565, Ibaraki, Japan
[3] Division of Molecular Medicine, School of Pharmaceutical Sciences, University of Shizuoka, 52-1 Yada, Suruga-ku, Shizuoka 422-8526, Japan
[4] Department of Agricultural and Life Sciences, Faculty of Agriculture, Shinshu University, 8304 Minami-minowa, Kami-ina, Nagano 399-4598, Japan
[5] Department of Biomolecular Innovation, Institute for Biomedical Sciences, Shinshu University, 8304 Minami-minowa, Kami-ina, Nagano 399-4598, Japan
* Correspondence: ttakaya@shinshu-u.ac.jp

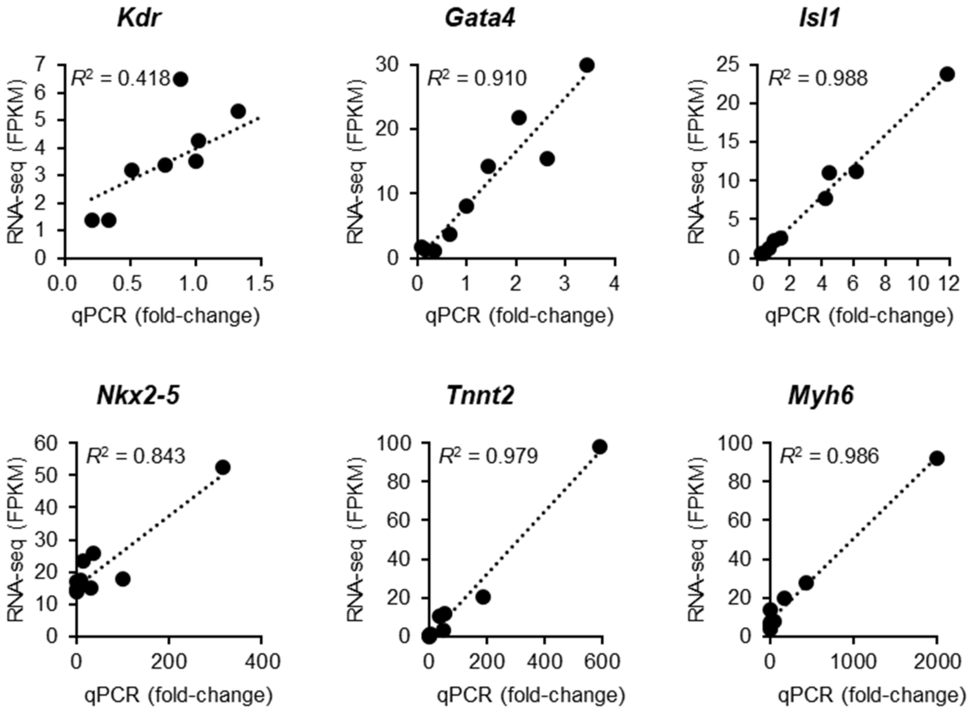

Supplementary Figure S1. Correlation of the gene expression levels quantified by RNA-seq and qPCR. The FPKM values defined by RNA-seq and the mRNA levels (fold-changes) detected by qPCR (Figure 2C) were analyzed using Pearson's correlation coefficient test.

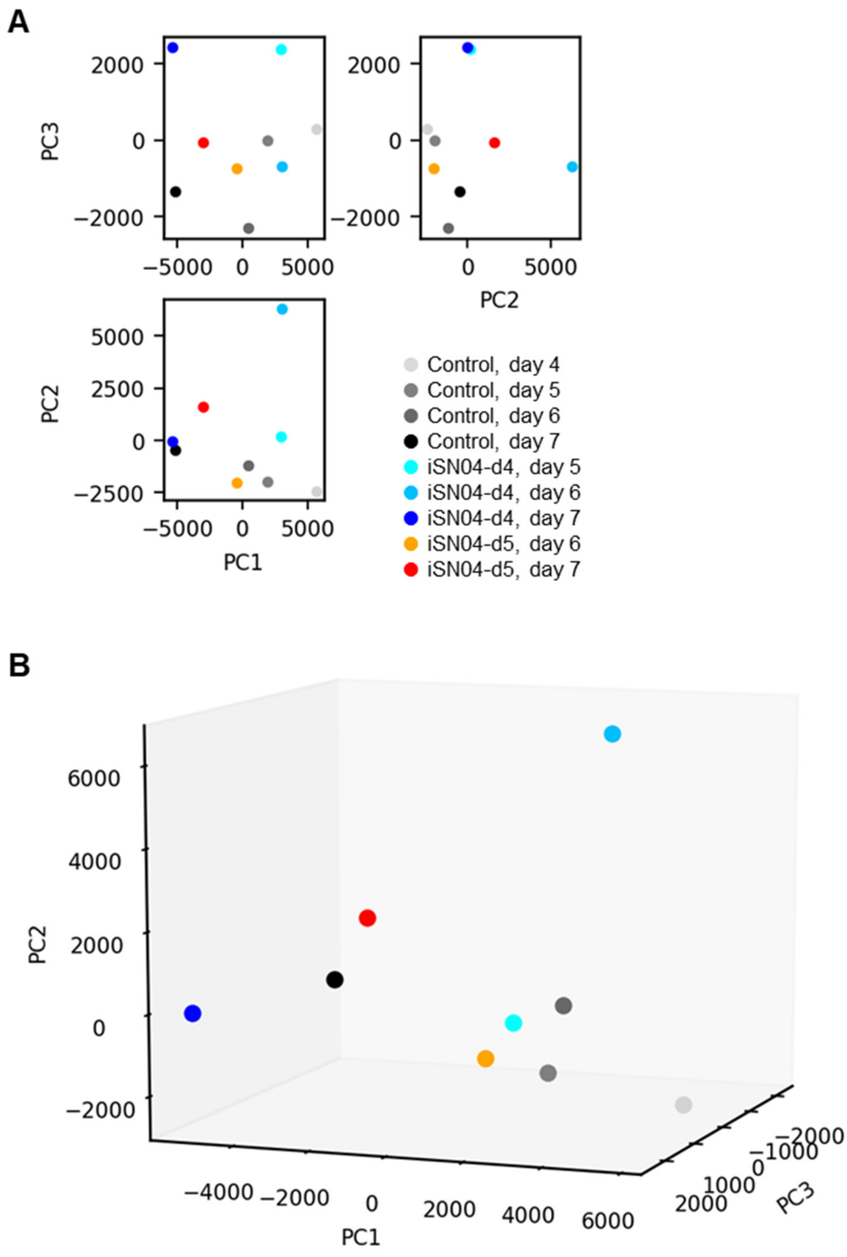

Supplementary Figure S2. PCA of the RNA-seq data. The 13,613 FPKM values obtained by RNA-seq of hCGp7 cells induced to differentiate in DM and treated with 10 μM iSN04 from day 4 or 5 on 30-mm dishes (same samples as in Figure 2C). (**A**) Two-dimensional plots on the PCA space reconstructed by PC1 (contribution, 0.52), PC2 (0.26), and PC3 (0.09). (**B**) Three-dimensional plot on the PCA space reconstructed by PC1, PC2, and PC3.

Supplementary Table S1. Primer sequences for qPCR.

Gene	Sequence (5′-3′)	Reference
Isl1	GAGTCATCCGAGTGTGGTTTC<br>ACCATGGGAGTTCCTGTCATC	[40]
Kdr	TGCAGACAGAAATACGTTTGAGAAC<br>GCAAACTGGTGTGAGTGATTCG	[41]
Klf4, endogenous	GCGAACTCACACAGGCGAGAAACC<br>TCGCTTCCTCTTCCTCCGACACA	[2]
Gata4	TCTCACTATGGGCACAGCAG<br>GCGATGTCTGAGTGACAGGA	[19]
Myh6	GAGATTTCTCCAACCCAG<br>TCTGACTTTCGGAGGTACT	[18]
Nanog, endogenous	CAGGTGTTTGAGGGTAGCTC<br>CGGTTCATCATGGTACAGTC	[2]
Nkx2-5	CAAGTGCTCTCCTGCTTTCC<br>GGCTTTGTCCAGCTCCACT	[18]
Pou5f1, endogenous	TCTTTCCACCAGGCCCCCGGCTC<br>TGCGGGCGGACATGGGGAGATCC	[2]
Sox2, endogenous	TAGAGCTAGACTCCGGGCGATGA<br>TTGCCTTAAACAAGACCACGAAA	[2]
Tnnt2	CAGAGGAGGCCAACGTAGAAG<br>CTCCATCGGGGATCTTGGGT	[42]
Ywhaz	TTGATCCCCAATGCTTCGC<br>CAGCAACCTCGGCCAAGTAA	[36]

Supplementary Video S1. Beating cardiomyocytes spontaneously differentiated from miPSCs. Undifferentiated 20D17 cells were seeded on gelatin-coated dishes and induced spontaneous differentiation in DM for 11 days.

Supplementary Video S2. Beating cardiomyocytes differentiated from miPSCs treated with iSN04. Undifferentiated 20D17 cells were seeded on gelatin-coated dishes and induced spontaneous differentiation in DM for 11 days. The cells were treated with 10 μM iSN04 from day 5 to day 9.
